# Importance of interlayer H bonding structure to the stability of layered minerals

**DOI:** 10.1038/s41598-017-13452-7

**Published:** 2017-10-16

**Authors:** Michele Conroy, Jennifer A. Soltis, Rick S. Wittman, Frances N. Smith, Sayandev Chatterjee, Xin Zhang, Eugene S. Ilton, Edgar C. Buck

**Affiliations:** 1 Energy and Environment Directorate, Pacific Northwest National Laboratory, Richland, WA 99354 USA; 2 Physical and Computational Sciences Directorate, Pacific Northwest National Laboratory, Richland, WA 99354 USA; 3 National Security Directorate, Pacific Northwest National Laboratory, Richland, WA 99354 USA

## Abstract

Layered (oxy) hydroxide minerals often possess out-of-plane hydrogen atoms that form hydrogen bonding networks which stabilize the layered structure. However, less is known about how the ordering of these bonds affects the structural stability and solubility of these minerals. Here, we report a new strategy that uses the focused electron beam to probe the effect of differences in hydrogen bonding networks on mineral solubility. In this regard, the dissolution behavior of boehmite (γ-AlOOH) and gibbsite (γ-Al(OH)3) were compared and contrasted in real time via liquid cell electron microscopy. Under identical such conditions, 2D-nanosheets of boehmite (γ-AlOOH) exfoliated from the bulk and then rapidly dissolved, whereas gibbsite was stable. Further, substitution of only 1% Fe(III) for Al(III) in the structure of boehmite inhibited delamination and dissolution. Factors such as pH, radiolytic species, and knock on damage were systematically studied and eliminated as proximal causes for boehmite dissolution. Instead, the creation of electron/hole pairs was considered to be the mechanism that drove dissolution. The widely disparate behaviors of boehmite, gibbsite, and Fe-doped boehmite are discussed in the context of differences in the OH bond strengths, hydrogen bonding networks, and the presence or absence of electron/hole recombination centers.

## Introduction

The unique properties of 2D layered materials such as graphene have led to an explosion of interest in these materials^1,2^. Layered materials do not possess 3D atomic bonding. Rather, they have strong in-plane chemical bonds and weak out-of-plane bonds, which allows them to be exfoliated into extremely high-aspect-ratio 2D nano-sheets with enormous surface area^3^. Many layered minerals are composed of structural units (sheets) held together via out-of-plane hydrogen bonds. However, the role of the hydrogen bond network in their stability and dissolution behavior is not well known.

Two of the most common aluminum (oxy) hydroxide (AOH) mineral phases in nature, boehmite (γ-AlOOH) and gibbsite (γ-Al(OH)_3_), are classified as layered materials and owe their structural stability to hydrogen bond networks. The geochemical cycling and relative stability of these two phases has attracted considerable interest, as aluminum toxicity affects plants, humans, and other animals^4–8^. They are also key components in the Bayer process, which uses dissolution/re-precipitation to purify and extract Al from bauxite ore deposits^9,10^, and in the treatment of Al-rich radioactive waste generated during the nuclear fuel cycle^11–14^. The structural units, or layers, are composed of aluminum hydroxide and oxy (hydroxide) chains (gibbsite and boehmite respectively) which extend along the a-axis. In contrast to micas, clays, and double hydroxides, both boehmite and gibbsite do not (normally) have layer charges arising from isomorphic substitutions; consequently the interlayer region is devoid of charge balancing ions and layer cohesion is not due to electrostatic forces. Rather, within the interlayer region a network of out-of-plane hydrogen bonds ensure the integrity of the layered structure^15,16^. Although, the precise positions of hydrogen atoms between the interlayers, which determines the space group, are still actively debated in the literature^17–21^, the hydrogen bonding network for boehmite and gibbsite are very different. This provides an opportunity for learning more about the role of hydrogen bonding in their stability and dissolution behavior by using techniques that specifically target the hydrogen bonding network, and in doing so, provide new insight into one of the major knowledge gaps in layered mineral reactivity: Why some mineral pairs, such and boehmite and gibbsite, with relatively similar structures and compositions, possess radically different solubility behaviors^12,22,23^.

Indeed, most investigations of AOH solubility to date have not explored the potential connection between the structure of hydrogen bond networks and dissolution. Instead, the traditional focus has been tracking the concentration of Al in solution under the assumption that dissolution always occurs by a simple corrosion mechanism, and changes in morphology are not taken into account. However, one study on evolving nanoparticle morphology by Peskleway *et al*.^24^ documented the retreat of monolayer steps on the gibbsite basal surface during dissolution using *in situ* liquid cell atomic force microscopy, one of the first suggestions that dissolution in an AOH mineral may not proceed directly via uniform corrosion of particles from sidewalls inward.

In this contribution, drawing on recent reports of hydrogen generation during irradiation of solid AOH^25,26^, we used electron beam irradiation in tandem with liquid cell electron microscopy (LCEM) as a tool to intentionally disturb the hydrogen bond networks of boehmite and gibbsite, while observing their dissolution behavior *in situ*. LCEM has advantages over other *in-situ* liquid cell microscopy techniques (e.g., optical microscopy) because of its high temporal and spatial resolution. Whereas in many LCEM studies irradiation from the electron beam results in undesirable effects that complicate interpretation^27–30^, here we use such effects to our advantage to target the hydrogen bonding network of both minerals. Our results show that intense electron irradiation induces boehmite to dissolve, where dissolution and delamination are strongly coupled. In contrast, gibbsite neither delaminated nor dissolved. These results are contrary to AOH behavior under conventional dissolution conditions, where gibbsite dissolves readily, but dissolution of boehmite requires extreme pH and temperature. Through a series of LCEM experiments controlling the energy of irradiation, concentration of radiolysis products, and using Fe(III)-doped boehmite we suggest that dissolution was caused by disruption of the hydrogen bonding network of boehmite which destabilized the layered structure, resulting in delamination followed by rapid dissolution. The stability of gibbsite and specifically the fact that it did not delaminate during irradiation suggests that its hydrogen bonding network is more robust under these conditions compared to boehmite. The fact that gibbsite expresses two distinct hydroxyl groups on its basal surface compared to only one for boehmite^17–21,31^, might give the hydrogen bonding network of gibbsite a greater capacity to accommodate radiation induced defects. The surprising stability of Fe-doped boehmite relative to un-doped boehmite draws attention to the role of electron/hole pairs in the dissolution process. In sum, the dissolution of boehmite in the LCEM is a complex, multi-step process that includes internal delamination of individual particles to form thin exfoliated layers, with thicknesses on the order of that documented for boehmite fibers^32^, prior to complete dissolution. These results lend new insight into the importance of interlayer hydrogen ordering with regard to the structural stability of layered minerals.

## Results

**Figure 1:**
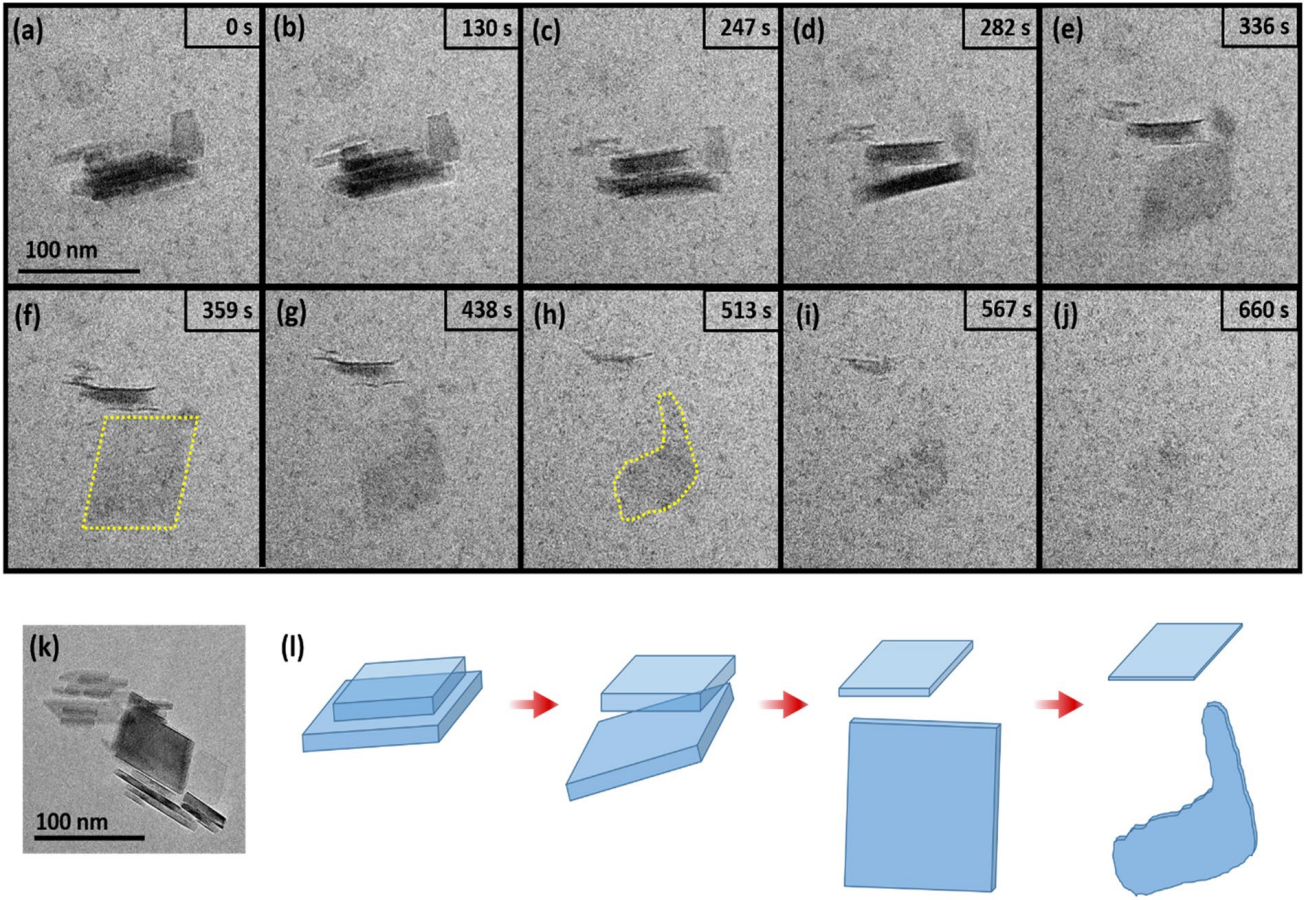
LCEM imaging of boehmite dissolution. (**a**–**j**) Sequential TEM images from Supplementary Movie 1, showing boehmite particles in deionized (DI) water under uniform TEM illumination (200 keV) resulting in the particles initially separating/delaminating and rotating, followed by dissolution, (**k**) stacking behavior of the boehmite also seen in cryo-TEM imaging of boehmite in water, (**l**) schematic representation of the delamination, rotating and dissolution steps. The “0 s” timestamp in (**a**) is an arbitrary reference point from which the elapsed time of later images can be compared (this area was also exposed to the electron beam during focusing prior to the acquisition of the “0 s” image).

When an aqueous suspension of boehmite was exposed to electron irradiation, the particles were observed to undergo rotational and translational movement, dissociate from each other, and ultimately—and unexpectedly—internally delaminate and dissolve. All of these behaviors are captured in Supplementary Movie 1. Selected still images and their corresponding timestamps are shown in Fig. 1. Except for the semi-detached particle on the upper right, boehmite is initially viewed close to edge on (i.e., parallel to the basal surface). The initial stacking nature of the boehmite aggregates was also documented in cryo-transmission electron microscopy (TEM) images of boehmite in deionized (DI) water (Fig. 1k), ensuring it was not an artifact of the dimensional confinement of the liquid cell.

As seen in Supplementary Movie 1, the stack of boehmite particles begins to split into three pieces after an induction period (Fig. 1c). We shall call this process “de-stacking” as the original particle appears to be composed of a stack of smaller particles that are presumably held together along their basal (010) surfaces by relatively weak forces. This de-stacking appeared to precede significant corrosion of the edges. By 336 s (Fig. 1e) the lower particle has rotated and presented its basal surface; it then begins to noticeably corrode from the sidewalls inward, losing its original defined rectangular shape (from ~438 s in Fig. 1g) and shrinking with continued beam exposure (from 438 s in Fig. 1g). In Supplementary Movie 1 one can see that the central particle, still edge-on, starts to shed or exfoliate thin layers in conjunction with corrosion of lateral surfaces. This process, which we shall call “delamination,” was captured in Fig. 1f,g and will be discussed in greater detail later. Dissolution was nearly complete by 660 s (Fig. 1j). Small (~5 nm) particles/dark regions were observed to form in the fluid cell sample after electron beam exposure.

A reasonable inference is that these secondary particles are scavenging aqueous Al from the dissolution of boehmite. We tested for this possibility by imaging a solution of 1 mM Al(NO_3_)_3_ under scanning transmission microscopy (STEM) conditions at 300 keV. Indeed, electron irradiation of the initial homogeneous solution induced the rapid formation of large Al-containing phases (>10 μm in diameter). A series of sequential STEM images of the Al(NO_3_)_3_ solution and particle formation is shown in Supplementary Fig. 1.

The dissolution of boehmite under the electron beam was quite unexpected, boehmite is known to be highly resistant to dissolution. Infrared (IR) spectroscopy indicates that OH stretching frequency is linearly and inversely related to OH bond strength^33–35^ and the interlayer OH groups in boehmite have lower OH stretching frequencies (bands at 3478, 3319, and 3129 cm^−1^) than for gibbsite (bands at ~3670, 3620, 3524, 3452, 3395, 3375, and ~3300 cm^−1^)^36,37^, indicating the interlayer OH bonds are weaker in boehmite. Conversely, lower interplanar OH stretching frequency also indicates stronger hydrogen^38–40^ bonding between layers, which may point toward an explanation for boehmite’s typical recalcitrance but does not explain the LCEM results. Consequently, we performed the same experiment on gibbsite to test the hypothesis that gibbsite would dissolve even more rapidly due to its weaker interlayer hydrogen bonding.

**Figure 2:**
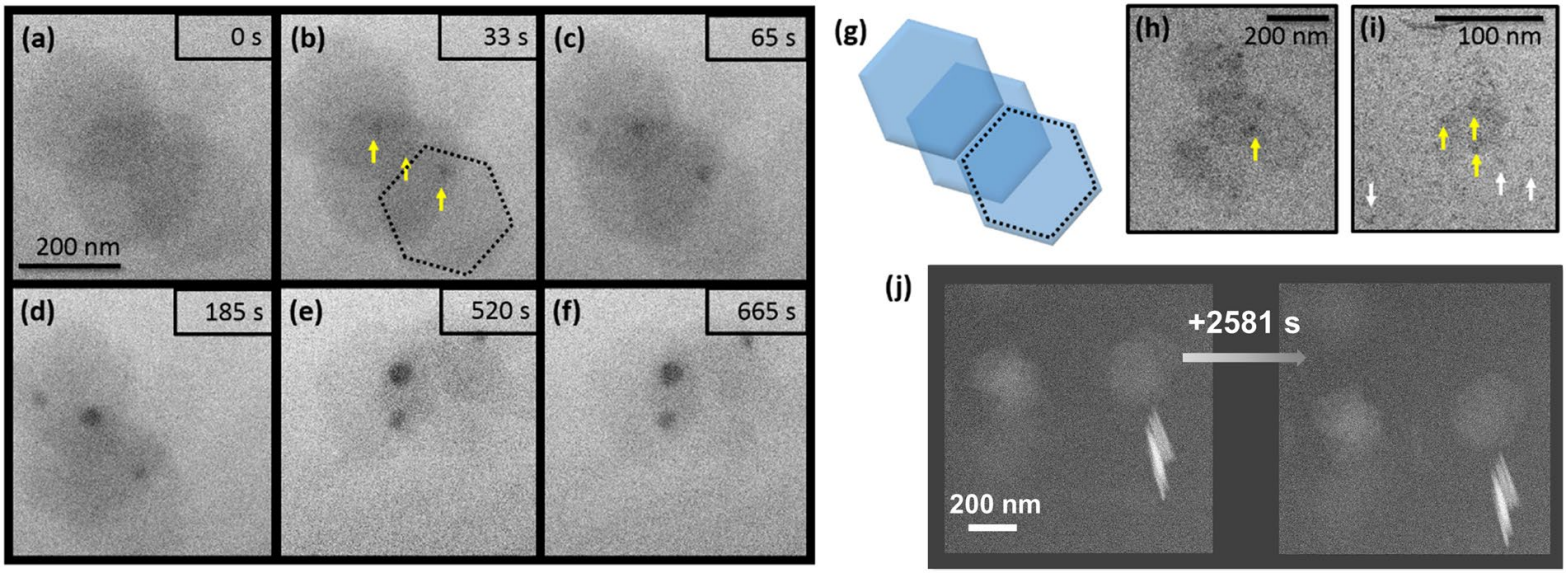
LCEM imaging of gibbsite in Deionized (DI) water under TEM and STEM illumination. (**a**–**f**) Sequential TEM images showing hexagonally shaped gibbsite particles in water rotating under uniform TEM illumination (200 keV) resulting in no measurable dissolution of the gibbsite and higher contrast nanoparticle growth on the gibbsite (indicated with yellow arrows), (**g**) schematic representation of the three assembled gibbsite particles imaged in (**a**–**f**), (**h**) TEM image of gibbsite particles after 1220 s of irradiation with yellow arrows highlighting the regions of higher contrast/nanoparticle growth, (**i**) digitally magnified region of Fig. 1(h) with yellow arrows highlighting the regions of higher contrast/nanoparticle growth on the boehmite plates during dissolution and white arrows highlighting the regions of higher contrast/nanoparticle growth away from the boehmite plates within the solution, (**j**) STEM images (300 keV).

Gibbsite nanoparticles did not dissolve under the electron beam when imaged under similar conditions (Fig. 2). Three overlapping gibbsite plates, shown in Fig. 2a–f, are drawn schematically in Fig. 2g. A dashed black line traces the perimeter of one of the gibbsite particles, which do not measurably change over time. The assemblage of particles was observed to rotate in solution and small, round dark spots began to appear on the basal surfaces (marked by yellow arrows in Fig. 2b). We note that STEM images of gibbsite in edge-on positions (lower right hand corner of Fig. 2h) show no signs of dissolution or delamination even after 2581 s of electron beam exposure. Therefore, the origin of the dark spots likely involves a very limited supply of labile surface Al.

The difference in dissolution behavior between boehmite and gibbsite was puzzling and contrary to expectation. Gibbsite is relatively easy to dissolve, while boehmite is well-known for its recalcitrance. We tested several hypotheses in order to probe these unexpected results:

**H1**. Radiolysis of the surrounding water creates a localized change in pH that causes boehmite to dissolve.

**H2**. Boehmite reacts with OH radicals or the reaction products of OH radicals formed via water radiolysis.

**H3**. Boehmite breaks down due to knock-on damage from the electron beam.

**H4**. The creation of electron/hole pairs destabilizes the hydrogen bond network and leads to dissolution.

Hypotheses 1–3 concern mechanisms that would be highly expected to affect gibbsite as well, and as shown in Fig. 2 did not. However, they involve reaction conditions and scenarios that are commonly observed in LCEM (**H1** and **H2**) or conventional TEM (**H4**) and must first be ruled out.

### H1. Localized change in pH due to radiolysis of water

The radiolysis of water produces many species, including H^+^ and OH^−^, and could cause localized changes in pH that contribute to boehmite dissolution. However, the fact that gibbsite did not dissolve as would be expected under extreme pH conditions supports the conclusion that there was no large localized pH change driving dissolution. Modeling of steady-state pH as a function of electron dose shows that the magnitude of the change in pH is, in fact, relatively minor in the context of the high pH values typically required for boehmite dissolution (on the order of pH 13–15) and, furthermore, that the pH decreases rather than increases^25,28^. The possibility that localized pH changes caused nanoscale differences in boehmite morphology and aggregation state was also tested experimentally by reacting boehmite with basic and acidic solvents (2 M KOH and 2 M HNO_3_ respectively) for 1 hr. at room temperature prior to vitrification and imaging using cryogenic (cryo) TEM. The duration and temperature of the reactions were chosen to mimic the conditions of the LCEM experiments. Cryo-TEM images in Supplementary Fig. 5 confirm the boehmite nanoparticles show no difference from boehmite suspended in DI water prior to vitrification (Fig. 1k).

**Figure 3:**
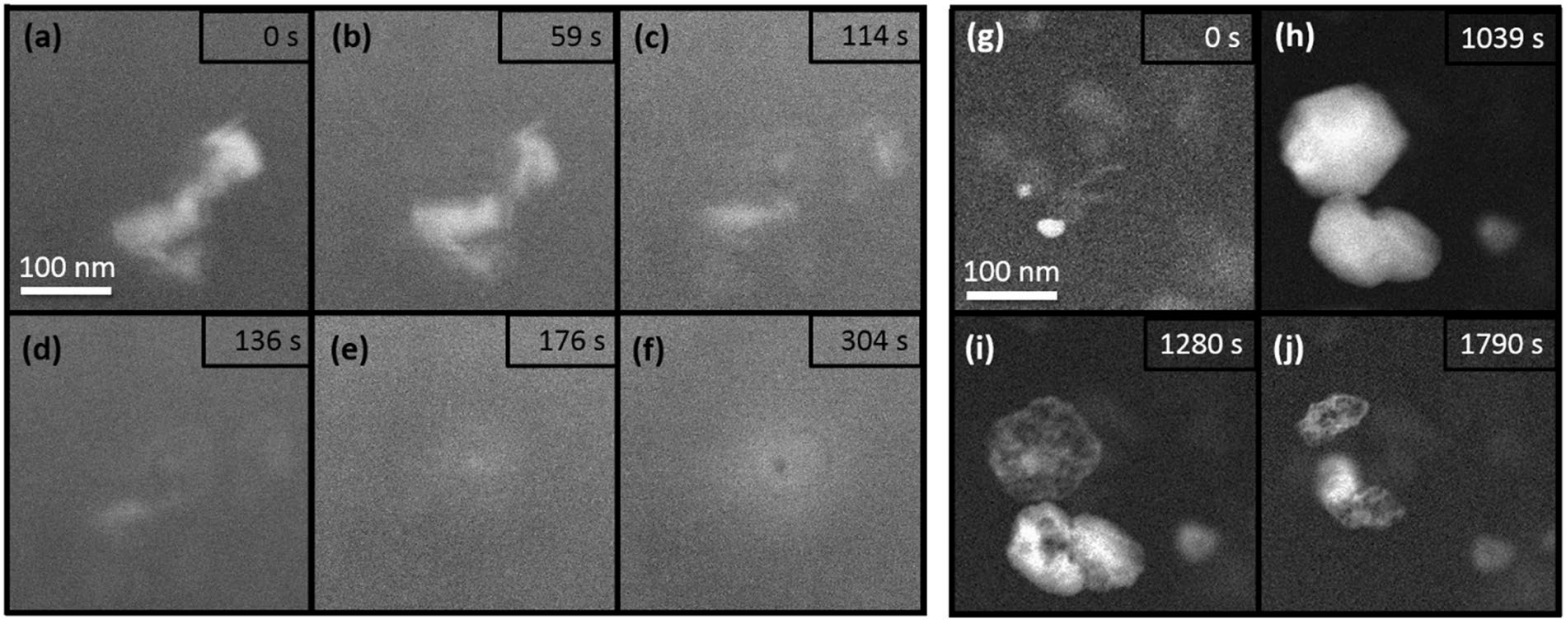
LCEM imaging in STEM mode of boehmite suspension in DI water. Images captured using (**a**–**f**) a 200 keV STEM probe and (**g**–**j**) a 300 keV STEM probe from Supplementary Movie 3.

The chosen imaging mode also plays an important role in the rate of radiolysis and subsequent effects such as changes in pH^27^. Both modeling and experiments showed that the use of a condensed STEM electron probe had a different effect than the use of evenly-spread electron beam illumination in TEM mode. Once again, there was no observation of dissolution when gibbsite was exposed to electron irradiation, this time at 300 keV in STEM mode (Fig. 2j). In contrast, imaging boehmite using STEM with a 200 keV (Fig. 3a–f and Supplementary Movie 2) and 300 keV (Fig. 3g–j) probe did cause the dissolution of boehmite particles, similar to TEM mode. Note that under 200 keV STEM illumination a cloudy spot formed by parking the probe in the center of the field of view. This was most likely due to the growth of a secondary aluminum phase (Fig. 3a–f and Supplementary Movie 2) as observed for imaging the AlNO_3_ solution in Supplementary Fig. 1. When imaged with the 300 keV STEM probe, the dissolution of boehmite was accompanied by the formation of relatively large precipitates (>100 nm) with distinct faceted edges, indicating that the structure was crystalline (Fig. 3g–j). The precipitates formed near or on a dissolving boehmite agglomerate after the first image was captured with a dwell time of 4 µs (bright areas of Fig. 3g). After growth (Fig. 3h), the particles began to decompose and shrink (Fig. 3i and j).

In sum, regardless of the illumination mode, boehmite dissolved but gibbsite did not. The stability of gibbsite is consistent with calculations indicating that pH stayed in the circumneutral region in both TEM and STEM mode. Nonetheless, the mode of radiation appeared to have a strong effect on the formation of a secondary phase. The formation of much larger secondary particles in STEM mode compared to TEM may possibly be explained by differences in the concentrations of radiolytic species produced. In contrast to TEM the STEM probe rasters across the sample, briefly exposing each small area of the sample to intense radiation. This highly localized electron dose can be sufficient to cause supersaturation conditions much faster than in TEM mode and result in nucleation of aluminum secondary phases from the dissolved Al species in solution. Modeling of continuous verses “pulsed” radiation exposure is discussed in the Supplementary Information. The calculated concentrations of radiolytic species for TEM and STEM conditions are shown in Supplementary Figs 4 and 5.

### H2. Reaction with OH radicals formed by water radiolysis

OH• is a highly reactive radiolytic species produced during aqueous LCEM experiments^26^. Bromine has long been used in the radiolysis field as an OH• scavenger. It consumes OH• radicals via the reaction:

1 $${{\rm{Br}}}^{-}+{\rm{OH}}\bullet \to {{\rm{BrOH}}}^{-}$$

**Figure 4:**
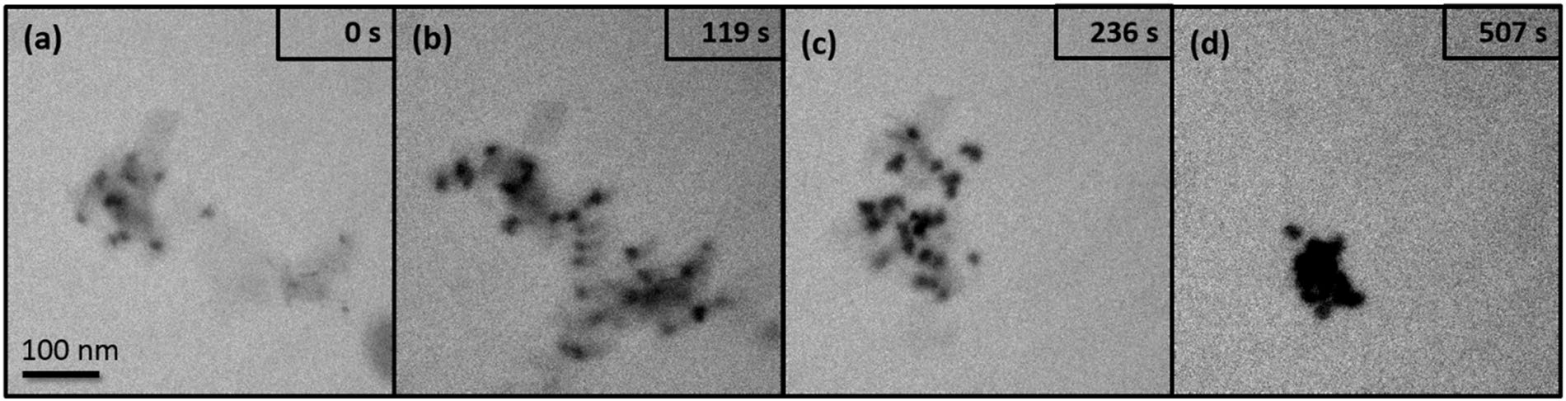
LCEM imaging of boehmite with radiolytic species scavengers. Boehmite in 10 mM KBr solution illuminated with a 200 keV TEM beam.

This reaction disables the subsequent reaction that converts OH• to H• and lowers the rate of H_2_O_2_ destruction. The simulated effect of Br^−^ on radiolytic yields with dose is shown in Supplementary Fig. 6 using the model developed by Wittman *et al*.^41^. The modeling results informed our decision to prepare a fresh suspension of boehmite in 10 mM KBr, as this concentration of Br^-^ would be expected to sufficiently scavenge the amount of OH• produced in our experiments. The imaging conditions were the same as in previous experiments. Supplementary Movie 3 and selected still images shown in Fig. 4 again report the rapid dissolution of boehmite, indicating that OH• and radiolysis products that result from the reaction of OH• were not responsible for boehmite dissolution. It is important to note that similar secondary phases of high contrast are formed during the dissolution as seen in Figs 1 and 3, supporting the observation that the dissolution of boehmite provided a supply of aqueous Al^3+^ that could be used to grow new particles.

### H3. Knock-on damage from the electron beam

The exclusion of extreme localized conditions in terms of pH and the hydroxyl radical as factors in boehmite dissolution focuses the following discussion on the potential effect of pervasive radiation interacting directly with the boehmite solids. This can be either by knock-on damage or ionization and the creation of electron/hole pairs. If so, this would indeed change the immediate cause of dissolution from “external” attack by water and hydroxyls at high pH (and radiolysis products) to an “internal” attack which could rationalize, although not yet explain, the reversed dissolution kinetics of boehmite and gibbsite under the electron irradiation compared to under high pH conditions. With respect to knock-on damage, one would expect higher energy radiation sources (e.g., 300 keV versus 200 keV) to cause more damage^42^; although Egerton and co-workers have stated that the displacement energy for H-atoms can be <1 eV. This would suggest that knock-on damage could be significant in these phases^43,44^. One test of the hypothesis that knock-on damage was not a driving factor for the dissolution of boehmite was the energy dependence of the effects. The boehmite dissolved more slowly when imaged in TEM mode at 300 keV than at 200 keV (Supplementary Fig. 8). However, as discussed in greater detail below, the fact that Fe-doped boehmite did not dissolve under identical imaging conditions, strongly suggests that knock-on or secondary knock-on effects are not involved in this instance. This supports the contention that knock-on displacement damage was not a major contributing factor to boehmite dissolution.

### H4. The creation of electron/hole pairs destabilizes the hydrogen bond network and leads to dissolution

**Figure 5:**
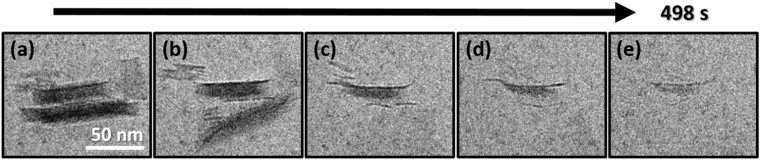
LCEM imaging showing the internal delamination of boehmite nanoparticles. Images (**a–e**) are cropped frames from Fig. 1 and Supplementary Movie 1.

When the boehmite dissolution process is observed in greater detail, it is clear that this is a multi-step process that departs from the classical dissolution models of corrosion from sidewalls inward or from pits on the nanoparticle surface. As seen in Supplementary Movie 1 and highlighted in Fig. 5, there is a close coupling between internal delamination of a single boehmite particle and its dissolution. Note that the very thin layers, ~1.2 to 2 nm thick (Supplementary Fig. 3), that were shed off the primary particles have lost rigidity and dissolve rapidly without further evidence of delamination. This is not surprising as such small particles are likely very unstable. It is worth commenting that their thickness is on the same order as the one-dimensional (1D) pseudo-boehmite fibers recently reported by Iijima *et al*.^32^, suggesting that the delaminated layers are possibly a similar form of pseudo-boehmite exfoliated from the bulk particle. High resolution TEM imaging and analysis of the dry boehmite down the (100) zone as seen in Supplementary Fig. 2 confirms that the average interlayer spacing between the two layers of double sheets (distance between the dark parallel lines) is ~0.6 nm, suggesting that the thin exfoliated layers are only 2/3 units thick.

The fact that dissolution and delamination are closely coupled for boehmite, but that gibbsite neither delaminates nor dissolves, strongly suggests that destabilization of the hydrogen bond network holding the structural units together is an integral part of the dissolution process, where the hydrogen bond network for boehmite and gibbsite are very different. In support of this hypothesis, Westbrook *et al*.^29^ observed the production of significantly more hydrogen release upon gamma irradiation of boehmite than gibbsite, pointing toward a greater ease of hydrogen removal from boehmite than from gibbsite. This is consistent with measurements of O-H stretch frequency from IR spectroscopy^45,46^ showing that the terminal O-H bond strength of gibbsite (3463–3468 cm^−1^) is stronger than that of boehmite (3290–3085 cm^−1^). The lower the wavenumber, the weaker the O-H bond but the stronger the H bonding between the structural units^29,30^. Kaddissy *et al*.^30^ suggested a more nuanced possibility where electron irradiation of both dry boehmite and bayerite (a polymorph of gibbsite) yielded hydrogen, where the results were dependent on crystallite size.

**Figure 6:**
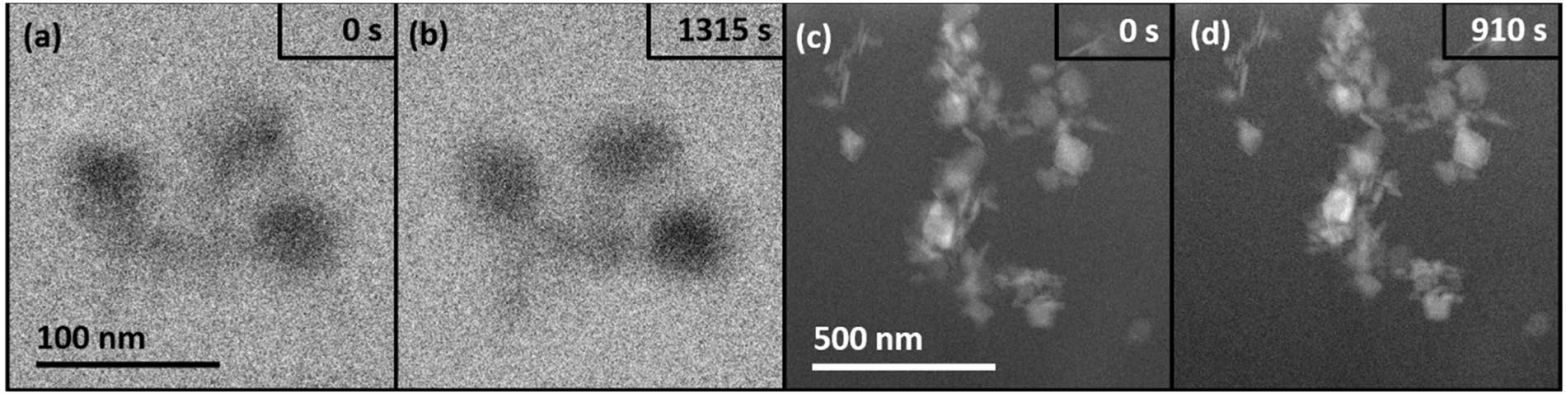
LCEM imaging of Fe-doped boehmite. (**a**) and (**b**) 200 keV TEM images and (**c**) and (**d**) 300 keV STEM images. Images (**c**) and (**d**) were acquired after an additional 1315 s (**c**) and 910 s (**d**) of electron beam exposure relative to images (**a**) and (**b**).

The role of interlayer hydrogen bonding was further investigated by altering the boehmite in such a way as to affect the terminal OH bond strength. Fe^3+^-doped boehmite (~1 wt% Fe) at the same crystallite size of the undoped boehmite, was prepared under the hypothesis that there would be a difference in the amount of energy required to remove hydrogen when the metal center was Fe rather than Al. Fe^3+^ was chosen because it has a similar size and charge as Al^3+^ (thus eliminating the need for any charge compensation)^47^. Strikingly, the Fe-doped boehmite imaged via LCEM under TEM (Fig. 6a,b) and STEM (Fig. 6c,d) mode show that the material was stable with no signs of dissolution or delamination, in sharp contrast to the undoped boehmite.

This discrepancy of solubility between boehmite, gibbsite and Fe-doped boehmite under the electron beam was interrogated via computational calculations to determine the amount of energy required to remove a proton from these structures. Single-molecule boehmite and gibbsite structures were developed after Westbrook *et al*.^29^ and tested alongside models in which Fe^3+^ was substituted for the Al center (Supplementary Fig. 11). Although our calculated binding energies are higher than in Westbrook *et al*., possibly due to the theoretical approach used, we observed the same trends when M = Al. It appears that there is a large difference in the ease of removing H from boehmite vs. gibbsite when Al^3+^ is the central atom in the cluster (i.e., about 5 eV easier to remove H from boehmite than from gibbsite), which may explain why gibbsite was not seen to dissolve even when boehmite was. However, when Fe^3+^ is substituted as the central atom in the cluster, the model predicted that it would be harder to remove H from boehmite; (Supplementary Table 1). Conversely, experimental IR data on γ-FeOOH^48^ suggests a weaker O-H bond than reported for γ-AlOOH which would suggest substituting Fe^3+^ for Al would weaken the O-H bond. Given the simplicity of the model but the low concentration of Fe^3+^ in the doped boehmite, it is not clear how to weight the present information. Interrogating the local bonding environment around Fe^3+^ (e.g., EXAFS) and performing complimentary larger scale modeling of boehmite doped at low Fe concentrations is warranted for future studies.

The creation of electron/hole pairs for both Al oxyhydroxides and Al hydroxides during electron irradiation was demonstrated by Kaddissy *et al*.^25^ When a hole is formed at the site of an oxygen atom, the reduced negative charge on the oxygen leads to a weakening of O-H bonds. Subsequent hydrogen loss would serve to compensate for the change in charge. As the loss of hydrogen from AOH minerals under irradiation has already been demonstrated^25,26^, it is possible that hydrogen loss or displacement occurs more frequently in boehmite than in gibbsite because the boehmite O-H bond is weaker. With enough events delamination might occur.

The hypothesis that electron/hole pair creation was the main cause for destabilizing the hydrogen bond network of boehmite is strengthened by the observation that Fe-doped boehmite did not dissolve. If Fe doping strengthens the OH bond, as suggested by our calculations, then one would indeed have expected the Fe doped boehmite to delaminate and dissolve more slowly than the un-doped boehmite, as observed. If on the other hand, Fe doping weakens the OH bond, as suggested by IR of lepidocrocite, then, all else equal, one would expect Fe-doped boehmite to be less stable than un-doped boehmite, the opposite of observations. If we go with the latter scenario, there has to be a compensating factor stabilizing Fe-doped boehmite. In this regard, Fe has been seen in other systems to serve as an electron trap (i.e., Fe^3+^ can reduce to Fe^2+^ in the TEM)^49,50^ and binary recombination center^51–53^ for electron/hole pairs. In the case of Fe-doped boehmite, Fe(III) might capture the ejected valence electron and reduce to Fe(II). Note that the Fe(III) does not have to be local to the ionization event, but could capture an ejected valence electron from a relatively distal oxygen. In fact, the non-local nature of this process increases its plausibility relative to strengthening of OH bonds: If doping increases the OH bond strength for hydroxyls associated with Fe, as our calculations suggest, and that is the only role for Fe, it is difficult to rationalize that 1% (the doping level) of stronger OH bonds associated with Fe will prevent destabilization of the H bond network. It is also possible that the Fe-doped boehmite could serve as a catalyst for recombining electron hole pairs in solution as Fe(III) could take up the solvated electron and then give it back to OH• or H_2_O_2_. In either case the Fe is serving as an electron trap that can neutralize a hole, whether that hole is in the solid structure or in solution, and the neutralization of this hole would therefore not lead to hydrogen loss or disruption of the H bonding network.

## Conclusions

The observation that delamination and dissolution of boehmite under electron beam irradiation was strongly coupled, but that gibbsite did not delaminate and dissolve, highlights differences in their H bond networks as a key factor controlling the relative stability of these layered minerals. Through a series of controlled experiments, we demonstrated that pH, radiolytic species, and knock on damage could not explain the different behavior of boehmite, gibbsite, and Fe-doped boehmite. Rather, the creation of electron/hole pairs appears to be the likely primary mechanism that is modified by several factors including the OH bond strength, the structure of the H-bond network, and the presence or absence of electron/hole recombination centers. A detailed theoretical effort is warranted to help untangle the possibilities, some of which have not yet been realized, but the present study has provided context and constraints that can serve as guideposts for future work. More generally, this study provides a better understanding of and appreciation for the importance of interlayer hydrogen bonding in layered mineral stability, as well as specific base-line information concerning the stability of Al (oxy)hydroxides in water during exposure to the electron beam.

## Methods

### Chemicals and materials

All chemicals were reagent grade or better. Aluminum nitrate nonahydrate (Al(NO_3_)_3_·9H2O; 98%) and aqueous ammonium hydroxide (NH_4_OH) solution (28% NH_3_ in H_2_O, ≥99.99% trace metals basis) were obtained from Sigma-Aldrich, USA. All solutions were prepared with DI water (18 MΩ resistivity).

### Boehmite and Gibbsite Synthesis

Rhombic boehmite plates on the order of 50–100 nm were prepared by a modification of literature procedures^14,54^, that involved a two-step process beginning with the alkaline hydrolysis of Al(NO_3_)_3_ to gibbsite, Al(OH)_3_, followed by the thermal dehydration of gibbsite to rhombic boehmite. 5.625 g Al(NO_3_)_3_.10H_2_O (0.015 M) was dissolved in 30 mL DI water at room temperature under stirring. NH_4_OH was added to the above mixture dropwise with stirring till the pH of the solution reached 10. Alkaline pHs resulted in the formation of a white precipitate, presumably indicating the formation of Al(OH)_3_. The overall volume of the reaction mixture was raised to 100 mL and stirring was continued for a further 15 minutes to form a uniform solution. The suspension was transferred to a Teflon-lined autoclave. The temperature of the resulting mixture was raised to 200 ± 2 °C and maintained at this temperature for 48 hours to yield a white solid. The mixture was allowed to cool, and the obtained solid was gravity-filtered, washed with excess water and methanol, air-dried and stored for further analyses. Fe-doped boehmite synthesis followed the same procedure as the pure boehmite except Fe(NO_3_)_3_•9H_2_O (1 wt% Fe relative to Al) was added to the solution and stirred prior to alkaline hydrolysis.

Gibbsite was prepared by using a hydrothermal method: Typically, Al(NO_3_)_3_·9H_2_O (≥98%, Sigma-Aldrich) was dissolved into deionized (DI) water under stirring to form a homogeneous solution with concentration 0.25 M at room temperature, followed by adding 1 M NaOH (≥98%, Sigma-Aldrich) aqueous solution to adjust the pH to around 5.0. After continuous stirring for 1 h, the solution was centrifuged to collect gel-like precipitations. The gel was washed with DI water three times. Gels were dispersed into DI water and then the solution was transferred to a 20 ml Teflon autoclave. The concentration of gels was 0.5 M and volume of the gel solution is 16 mL. The Teflon autoclave was sealed into a Parr bomb and was heated in electric oven at 80 °C for 3 days. The resulting white product was recovered by centrifuging and washing with DI water three times. The solid sample obtained was dried in oven at 80 °C overnight. Samples were characterized by various techniques including XRD, SEM, and TEM.

### Solid Phase Characterization

X-ray diffraction (XRD): XRD patterns of the samples were recorded on a Philips X’pert Multi-Purpose Diffractometer (MPD) (PANAlytical, Almelo, The Netherlands) equipped with a fixed Cu anode operating at 45 kV and 40 mA. XRD patterns were collected in the 5–100° 2θ-range with 0.04° steps at a rate of 5 s/step. Phase identification was performed using JADE 9.5.1 from Materials Data Inc., and the 2012 PDF4+ database from International Center for Diffraction Data (ICDD) database^55^. Patterns of the initial material identified pure highly crystalline boehmite, with no detectable pseudo boehmite, and pure gibbsite (Supplementary Figs 9 and 10). The Fe doped boehmite also only showed pure boehmite, but with some subtle differences. Un-doped boehmite had lattice parameters ***a*** = 2.8676, ***b*** = 12.2156, and ***c*** = 3.6945 Å, respectively. Doping with Fe increased the lattice parameters by 0.0023(***a***), 0.0063(***b***), and 0.0018(***c***) Å, close to but greater than our detection limit. Analysis of anisotropic line broadening indicated that the crystallite sizes of the Fe-doped and un-doped boehmite were similar in all three lattice directions.

Cryogenic transmission electron microscopy (cryo-TEM) samples were prepared by suspending boehmite nanoparticles in 2 M KOH, 2 M HNO_3_, or DI water at a concentration of 1 mg nanoparticles/mL solvent. The resulting suspension was sonicated for five minutes and allowed to equilibrate for one hour at room temperature. Samples were then used as-prepared or after aging in Nalgene bottles at 80 °C. Cryo TEM specimens were prepared by placing 3 μL of suspension onto a 200 mesh copper TEM grid coated with lacey carbon. The grids were cleaned in a Pelco EasyGlow glow discharger for one minute at 15 mA before using. The prepared grids were then blotted with filter paper for one second and plunged quickly into liquid ethane using a Vitrobot Mark IV (FEI, Hillsoboro, OR). Specimens were transferred to grid storage boxes under liquid nitrogen. All further handling was performed under liquid nitrogen to maintain cryogenic conditions. The vitrified specimens were loaded into a Gatan 626 cryo-TEM holder and imaged on an FEI Tecnai T20 TEM equipped with a field emission gun and operating at 200 keV. Images were acquired using TIA software (FEI, Hillsoboro, OR) on an FEI Eagle charge capture device (CCD) camera.

### Liquid cell electron microscopy

LCEM imaging was performed on suspensions of boehmite or gibbsite nanoparticles at 0.5 mg/mL in ACS-grade deionized water. Additional suspensions of boehmite at 0.5 mg/mL in 10 mM KBr were also prepared. The suspensions were sonicated for five minutes before loading into a Hummingbird fluid cell TEM holder with electrobiasing capabilities. The TEM holder was equipped with silicon nitride chips with 50 μm by 200 μm windows (Hummingbird Scientific). The window membrane was 50 nm thick. Chips were plasma cleaned (Harrick Plasma cleaner) under a 10% O_2_/90% Ar gas mix for 5 minutes on low prior to assembly of the fluid cell TEM holder. A 4 μL drop of the suspension was placed onto a 100-nm parallel spacer chip (window parallel to spacer). A spacer-free chip was then placed on top with the windows oriented perpendicular to each other during holder assembly.

After assembly, the holder was primed by flowing the suspension for 30 minutes at 50 μL/min and tested for leaks under high vacuum. The holder was transferred to the electron microscope. Microscopes used were a T20 TEM (described above) operated at 200 keV in TEM and STEM modes, a Titan 80/300 TEM/STEM (FEI, Hillsboro, OR) operated in STEM mode at 300 keV, and a Titan ETEM (FEI, Hillsboro, OR) operated at 300 keV. Imaging was done while the boehmite suspension was flowing at 5 μL/min. All specimens were irradiated with an electron dose of 36 ± 2 e^−^/Å^2^ as measured at the phosphor screen unless otherwise indicated.

### Modeling

Radiolysis generation can be predicted using the commercial software packages, FACSIMILE (MPCP Software Ltd., Faringdon, UK)^56,57^ and MAKSIMA-CHEMIST (Atomic Energy of Canada, 1979)^58–60^ to determine the concentrations of radiolytic species generated from a large number of rate equations with individual rates that vary by several orders of magnitude, necessitating stiff ordinary differential equation (ODE) solvers. In the case of pure water there are 73 equations and 15 radiolytic products to balance. The addition of Br^-^ ions, CO_2_, or Al^3+^, as well as the exceptionally high doses encountered in the electron microscope, will significantly increase the complexity. As the kinetic rate equations for these systems are not always well-known, the results may only be guidelines to the actual systems behavior. In this investigation, we used a diffusional-radiolytic model developed by Wittman *et al*.^41^ that uses the ODEPACK (Lawrence Livermore National Laboratory) stiff solvers and also the highly efficient model developed by Schneider^28^ *et al*. that uses Matlab ode stiff solvers.

## Electronic supplementary material

Supplementary Information

Supplementary Movie 1

Supplementary Movie 2

Supplementary Movie 3
