# Supplementary material for: Importance of interlayer H bonding structure to the stability of layered minerals
Source: Sci Rep. 2017 Oct 16;7:13274. doi: 10.1038/s41598-017-13452-7 (PMC5643302; doi:10.1038/s41598-017-13452-7)
Supplement: Supplementary file 1 — Supplementary Information [file 41598_2017_13452_MOESM1_ESM.pdf]

Importance of interlayer H bonding structure to the stability of layered minerals

Supplementary Information

Michele Conroy[1]*, Jennifer A. Soltis[2]*, Rick S. Wittman[3], Frances N. Smith[1], Sayandev Chatterjee[1], Xin Zhang[2], Eugene S. Ilton[2], Edgar C. Buck[1]

1. Energy and Environment Directorate, Pacific Northwest National Laboratory, Richland, WA, 99354, USA
2. Physical and Computational Sciences Directorate, Pacific Northwest National Laboratory, Richland, WA, 99354, USA
3. National Security Directorate, Pacific Northwest National Laboratory, Richland, WA, 99354, USA

* These authors contributed equally to this work.
Correspondence and requests for materials should be addressed to E.C.B. (email: edgar.buck@pnnl.gov)

Table of Contents

Modeling production of radiolytic species under TEM and STEM conditions............................. 1
Captions for Supplementary Movies 2
Supplementary Figures 3
Supplementary Table 8
References... 9

Modeling production of radiolytic species under TEM and STEM conditions

Electron microscope images were recorded with a Titan 80/300 TEM/STEM (FEI) at 300 keV and with an FEI Tecnai TEM at 200 keV. We adopted the methods for estimating the electron dose based on de Jonge et al.[1] The dose based on value reported on the phosphor screen was 3.6×10^9 electrons/μm^2. For TEM and STEM imaging, the illuminated sample area was approximately 1 μm^2.

In TEM mode the dose builds gradually over time. At around 400 secs, when significant changes were observed in the boehmite-H_2O system, the OH radical concentration had reached a steady-state condition (Supplementary Fig. 4). However, in STEM, the beam rasters across the

specimen, as the beam sweeps across the region, effectively acting as a pulsed system where the length of the pulse is the dwell time (~2 µsec) and the interval with no irradiation is the total time for a scan (1-2 sec).

Supplementary Fig. 5 shows that the general trend of radiolytic species production under STEM conditions is the same as for the continuous irradiation shown in Supplementary Fig. 4 for TEM imaging; however, because the dose is stronger, the steady state condition is approached faster. In terms of impact to a chemical system, the drastic localized change in pH may have the greatest impact on inducing precipitation or dissolution.

The stationary STEM probe causes the most change owning to its much greater intensity over the much weaker and dispersed TEM beam. However, the rastering effect of the beam in STEM can create localized pH changes that would be more severely impacting to an in-situ liquid cell experiment than the TEM beam.

Captions for Supplementary Movies

Supplementary Movie 1. TEM movie of boehmite nanoparticles in deionized (DI) water imaged at 200 keV. A stack of boehmite nanoparticles is positioned nearly perpendicular to a lateral surface. Over the first 300 s, the particles begin to “de-stack” and separate from each other. One rotates such that the rhombohedral shape of a boehmite plate is visible and corrodes from the sidewalls inward over an additional ~400 s. Simultaneously, another particle undergoes internal delamination, maintaining an orientation perpendicular to a lateral surface while shedding thin layers. These layers distort and dissolve over the following ~400 s.

Supplementary Movie 2. STEM movie of boehmite nanoparticles in deionized (DI) water imaged at 200 keV. An agglomerate of boehmite particles rotates and dissolves over the course of 176 s. By the end of the dissolution process, a cloudy area has formed in the center of the field of view where the beam was parked and continues to grow in intensity until imaging ceased at 304 s.

Supplementary Movie 3. TEM movie of boehmite nanoparticles in 10 mM KBr imaged at 200 keV. An agglomerate of boehmite nanoparticles dissolves rapidly over the first few hundred s and no rhombohedral particles are visible at 507 s. Dark spots ~30 nm in diameter began forming almost immediately in close spatial proximity to the boehmite plates. The secondary particles increased with number over time until they were the only phase remaining.

Supplementary Figures

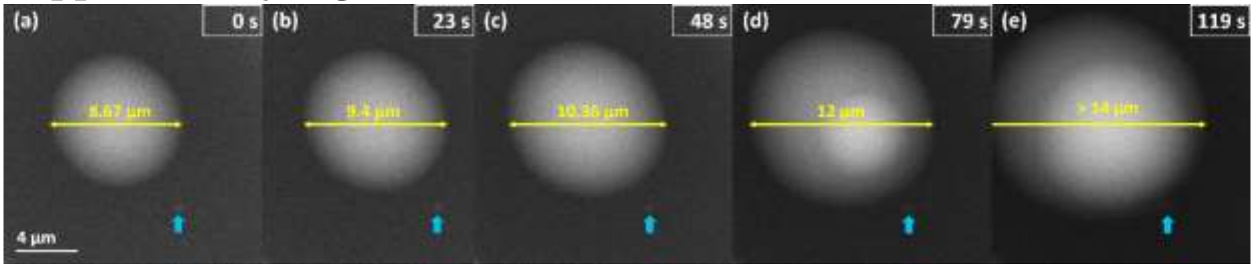

Supplementary Figure 1. STEM images of a solution of 1 mM $AlNO_3$ in DI water acquired using a 300kV probe.

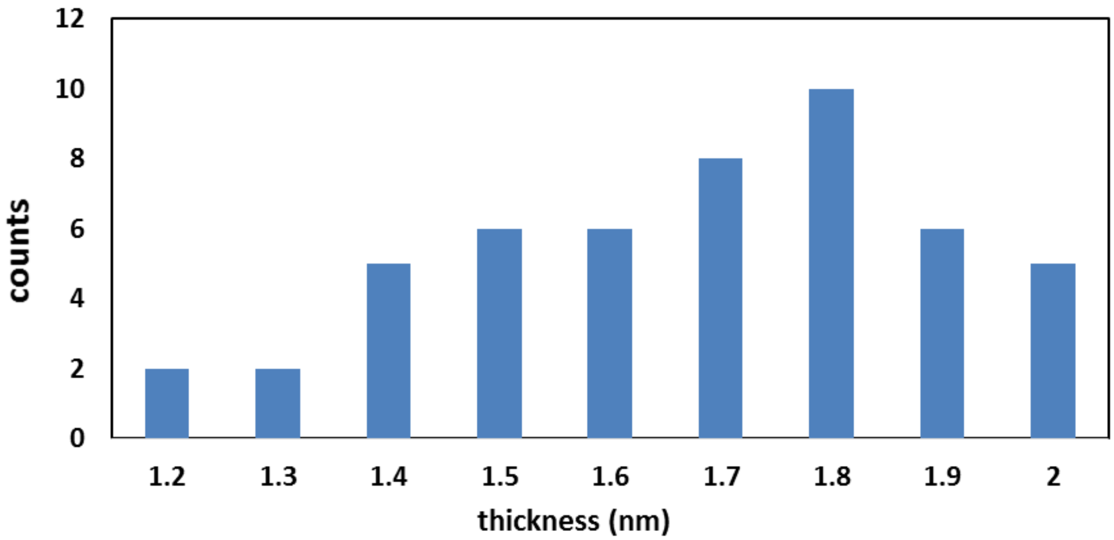

Supplementary Figure 2. Thickness distributions of delaminated thin layers measured from figures seen in Figure 2 of the main article and Supplementary Movie 1. The histogram of thickness distributions reveals a peak centered at 1.7–1.8 nm, which corresponds to the width of 2-3 layers of the Al–O octahedral double sheet.

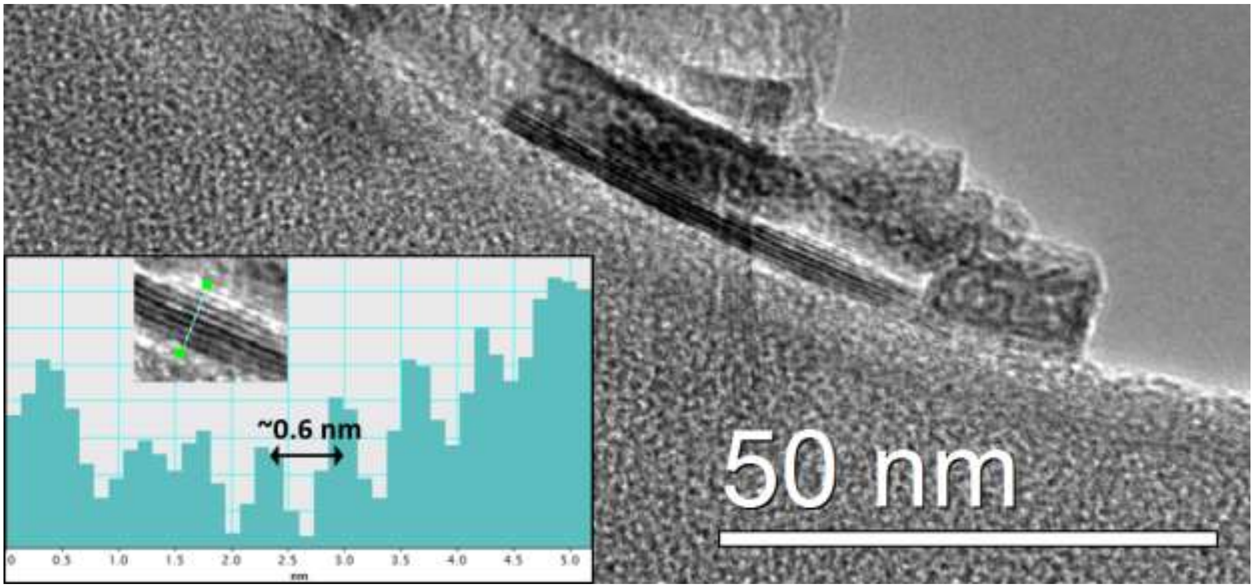

Supplementary Figure 3. High resolution TEM images of dry boehmite in vacuum, histogram measuring the lattice spacing along the (020) zone.

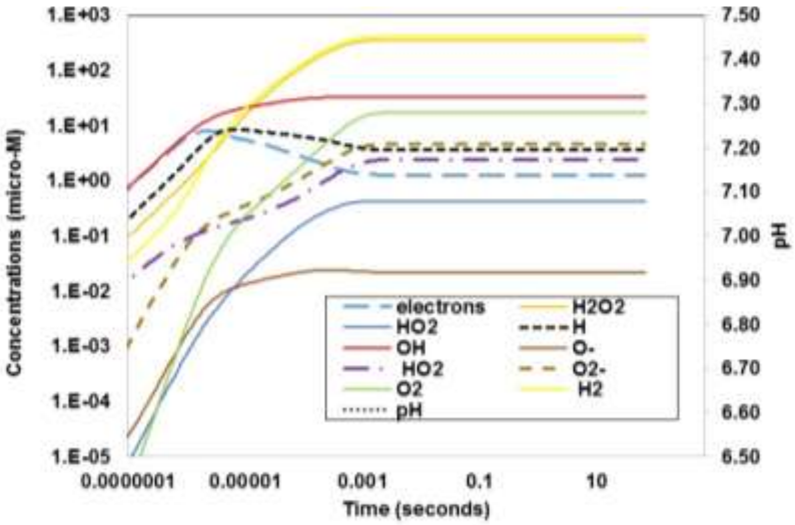

Supplementary Figure 4. Plot showing the development of reactive radiolytic products over 660 seconds under TEM conditions. The •OH radical is the highest concentrated species. Calculations based on the estimated volume irradiated and the concentration (reported in µM) of species in this volume.

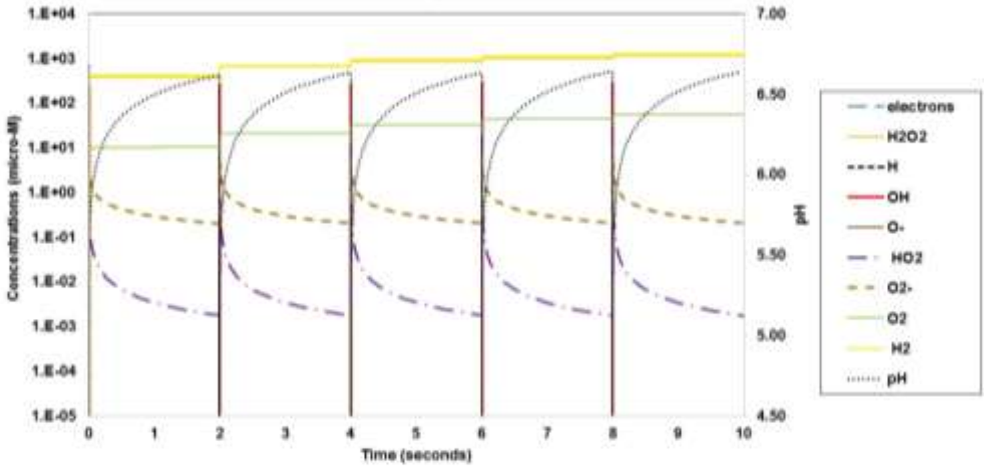

Supplementary Figure 5. Plot showing the development of reactive radiolytic products over 10 seconds under STEM conditions. The •OH radical spikes during each scan and quickly dies away as do other radicals. There is also a sudden decrease in pH at each spike. The pH change is much greater because the STEM probe is a condensed TEM beam and is at least 100× brighter.

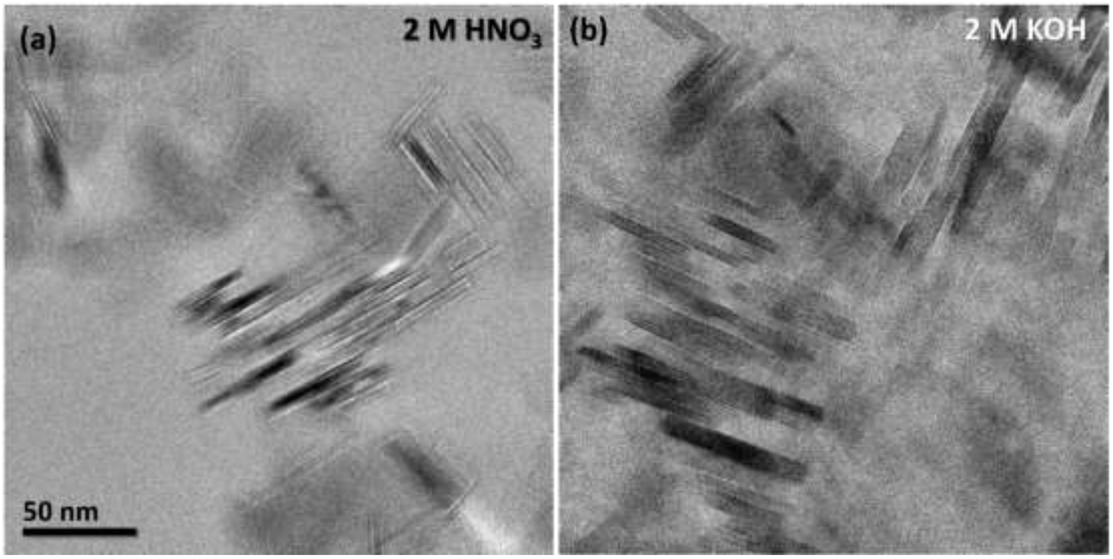

Supplementary Figure 6. Cryo-TEM images of Boehmite vitrified after 1hr suspended in a (a) 2 M KOH solution and (b) 2 M HNO_3 solution.

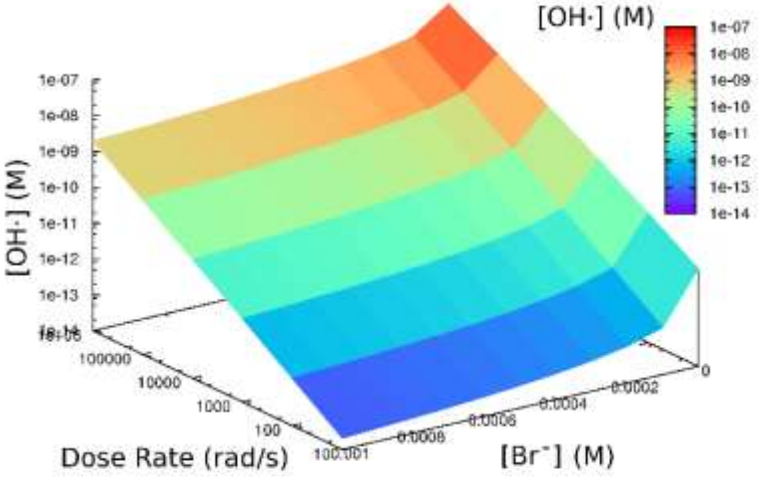

Supplementary Figure 7. Predicted effect of Br^- on the OH• concentration as a function of beta-gamma dose rate.

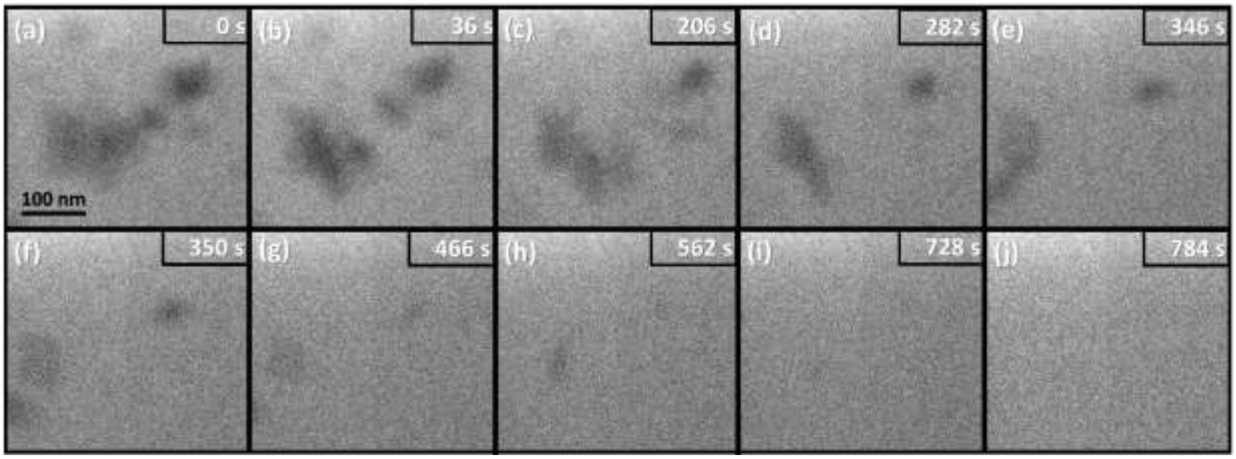

Supplementary Figure 8. TEM images of an aqueous suspension of boehmite in a liquid cell imaged at 300 keV.

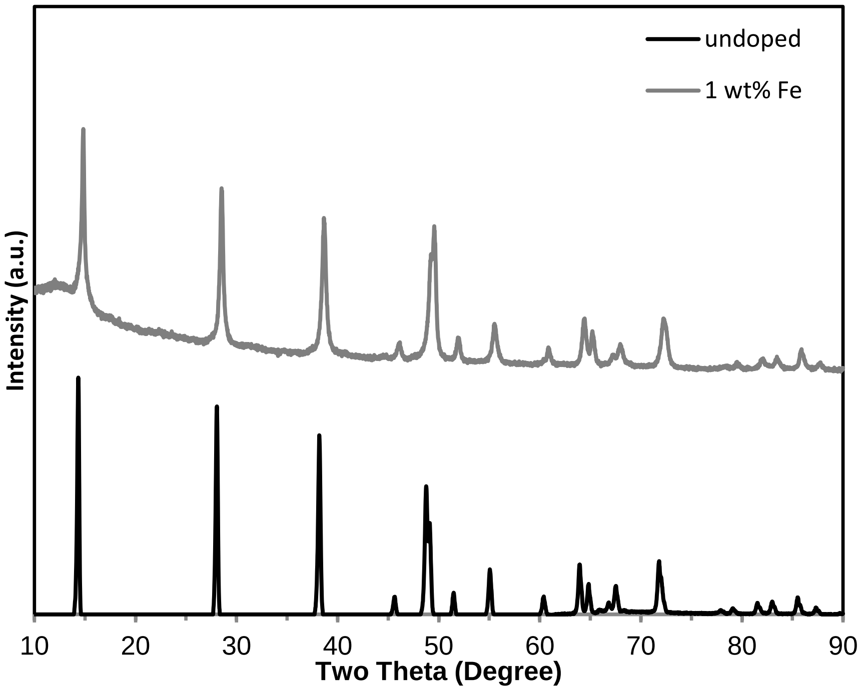

Supplementary Figure 9. XRD patterns of undoped and 1 wt% Fe-doped boehmite. Patterns have been vertically offset for clarity.

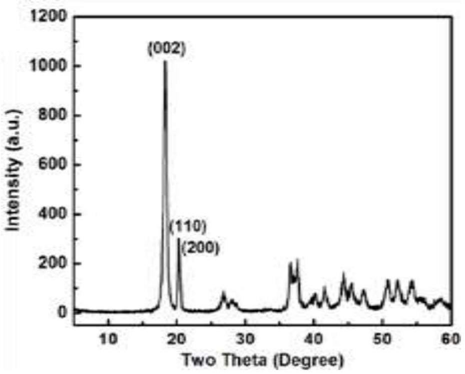

Supplementary Figure 10. XRD pattern of gibbsite nanoparticles.

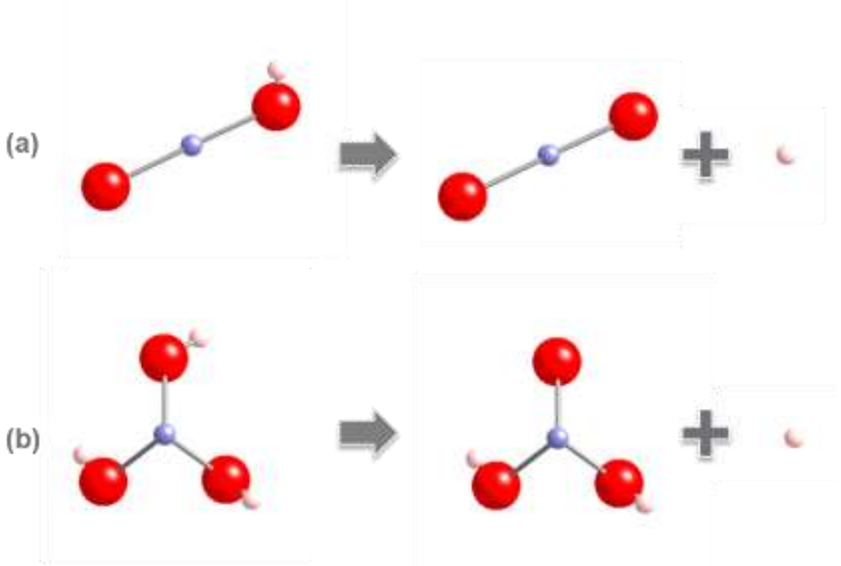

Supplementary Figure 11. Ball and stick models of (a) boehmite-like and (b) gibbsite-like molecules. Charge neutral (left of arrow) and defect clusters (right of arrow) represent models used in calculating deprotonation energies. Purple spheres are Al^{3+}, red spheres are O^{2-}, and white spheres are H^+.

Supplementary Table

Model	E_{clus} (H_a)	E_{def} (H_a)	E_{H^+} (H_a)	E H bond (H_a)	E H bond (eV)
Boehmite Model (Al) - H removed	-392.1681	-391.7062	0.01*	0.4719	12.84
Gibbsite Model (Al) - H removed	-468.5206	-467.8874	0.01*	0.6432	17.50
Boehmite Model (Fe) - H removed	-1412.7355	-1412.0901	0.01*	0.6554	17.84
Gibbsite Model (Fe) - H removed	-1488.8939	-1488.2686	0.01*	0.6353	17.29

Supplementary Table 1. Calculated values of the energy of the cluster (E_{clus}), deprotonated cluster (E_{def}), proton (E_{H^+}), and hydrogen—cluster bond (H bond).

*A value of 0.01 H_a is applied since one cannot calculate the energy of H^+ directly. (Tawa et al. 1998)[2]

References

1 de Jonge, N. & Ross, F. M. Electron microscopy of specimens in liquid. *Nat. Nanotechnol.* **6**, 695-704, doi:10.1038/nnano.2011.161 (2011).

2 Tawa, G. J., Topol, I. A., Burt, S. K., Caldwell, R. A. & Rashin, A. A. Calculation of the aqueous solvation free energy of the proton. *J. Chem. Phys.* **109**, 4852-4863, doi:10.1063/1.477096 (1998).
